# Key Technology Considerations in Developing and Deploying Machine Learning Models in Clinical Radiology Practice

**DOI:** 10.2196/28776

**Published:** 2021-09-09

**Authors:** Viraj Kulkarni, Manish Gawali, Amit Kharat

**Affiliations:** 1 DeepTek Inc Pune India; 2 D Y Patil University Pune India

**Keywords:** artificial intelligence, AI, machine learning, deep learning, radiology, privacy, neural networks, deployment

## Abstract

The use of machine learning to develop intelligent software tools for the interpretation of radiology images has gained widespread attention in recent years. The development, deployment, and eventual adoption of these models in clinical practice, however, remains fraught with challenges. In this paper, we propose a list of key considerations that machine learning researchers must recognize and address to make their models accurate, robust, and usable in practice. We discuss insufficient training data, decentralized data sets, high cost of annotations, ambiguous ground truth, imbalance in class representation, asymmetric misclassification costs, relevant performance metrics, generalization of models to unseen data sets, model decay, adversarial attacks, explainability, fairness and bias, and clinical validation. We describe each consideration and identify the techniques used to address it. Although these techniques have been discussed in prior research, by freshly examining them in the context of medical imaging and compiling them in the form of a laundry list, we hope to make them more accessible to researchers, software developers, radiologists, and other stakeholders.

## Introduction

Although radiology imaging has emerged as an indispensable tool in diagnostic medicine, there is a worldwide shortage of qualified radiologists to read, interpret, and report these images [1,2]. The volume of images is growing faster than the number of radiologists. The high workload that this causes leads to errors in diagnosis because of human fatigue, unacceptable delays in reporting, and stress and burnout in radiologists. On the other hand, artificial intelligence (AI) and machine learning models have shown remarkable performance in the automated evaluation of medical images [3-5]. In this situation, hospitals are increasingly drawn toward adopting computer-aided detection technologies for processing scans. These technologies show considerable promise in improving diagnostic accuracy, reducing reporting time, and boosting radiologist productivity.

Supervised machine learning, the most common form of machine learning, works in two phases. In the first phase, the algorithm implemented as a software reads a training data set consisting of images along with their corresponding labels. It processes these data, extracts patterns from it, and learns a function that maps an input image to its corresponding label. The learned mapping function along with the extracted patterns are mathematically represented in the form of the trained model. This is called the *training* phase. In the second phase, called the *inference* phase, the trained model is used to read input images and make predictions. Artificial neural networks are a class of machine learning algorithms; artificial neural networks with many layers are called deep neural networks. In the literature, the terms deep learning, AI, and artificial neural networks tend to be used interchangeably. In this paper, we use *machine learning* to broadly refer to all the terms mentioned earlier in addition to conventional machine learning algorithms, such as linear regression, support vector machines, decision trees, and random forests.

The development of machine learning models for radiology involves many challenges. High-quality training data are vital for good model performance [6] but are difficult to obtain. Available data may lack volume or diversity. It may be scattered across multiple hospitals. Even if the image data are available, they may not be labeled. Radiology scans suffer from a high degree of interreader variability, where 2 or more radiologists label the data inconsistently [7,8]; this may lead to noise or uncertainty in the ground truth labels. The distribution of target classes may be heavily skewed, especially for rare pathologies. This imbalance in class representation is often accompanied by unequal misclassification costs across classes. Care must be taken when dealing with imbalanced data sets, and this sometimes requires using special performance measures [9]. A model that works well on data from one hospital may perform poorly on data from a different hospital [10]. Similarly, a model deployed in practice at a hospital may experience a gradual decay in performance at the same hospital [11]. Machine learning models have been shown to be vulnerable to malicious exploits and attacks [12-14]. To support adoption by radiologists, the deployed models should be able to explain their decisions [15], and they should not discriminate patients on the basis of gender, ethnicity, age, income, among others [16].

This study has a simple structure. In the Key Considerations section, we enumerate the key considerations that machine learning researchers should acknowledge and address. For each consideration, we describe the common challenges and their significance before suggesting solutions to overcome them. In the Conclusions section, we discuss other overarching limitations that hinder the adoption of machine learning in clinical radiology practice.

## Key Considerations

### Insufficient Training Data

Machine learning models are data hungry, and their performance depends heavily on the characteristics of the data used to train them [6]. The training set size has a direct and significant effect on the performance of the models. However, the heterogeneity and diversity of the training data influence the ability of the models to generalize to unseen data sources [17]. To develop robust machine learning models, researchers need access to large medical data sets that adequately represent data diversity in terms of population features such as age, gender, ethnicity, and medical conditions and imaging features such as equipment manufacturers, image capture settings, and patient posture. Most available data sets in medical imaging do not meet these requirements [18-20]. As many critical conditions have low rates of occurrence, very little data are available for them. Machine learning models trained using these scanty data to diagnose rare conditions fail to perform well in practice even if they demonstrate good performance in retrospective evaluations.

Several methods have been proposed for dealing with insufficient data for training models. Data augmentation techniques including geometric transformations and color-space transformations can enhance the quantity and variety of training data [21]. Generative adversarial networks have shown success in generating synthetic images for rare pathologies, which can be further used for model training [22]. Although these techniques allow models to be trained on scarce data by artificially increasing the variation in the data set, they cannot serve as a substitute for high-quality data.

### Decentralized Data Sets

Many medical data sets are naturally distributed across multiple storage devices connected to networks owned by different institutions. In traditional machine learning settings, these data sets need to be consolidated into a single repository before training the models. Moving large volumes of data across networks poses several logistical and legal challenges [23]. Government policies such as the General Data Protection Regulation [24], the Health Insurance Portability and Accountability Act [25], and the Singapore Personal Data Protection Act [26] also stipulate restrictions on sharing and movement of data across national borders.

Privacy-preserving distributed learning techniques such as federated learning [27] and split learning [28] enable machine learning models to train on decentralized data sets at multiple client sites without moving the data and compromising privacy. Implementing these techniques, however, entails additional overheads, which may render the exercise unfeasible. These overheads include the high cost of developing software that supports these technologies, the high network communication bandwidth required, the orchestration effort in deploying it at multiple sites, and the possibly reduced performance of the predictive models [29]. Federated learning generates a global shared model for all clients, leading to situations where, for some clients, the local models trained on their private data perform better than the global shared model. In such situations, additional personalization techniques may be required to fine-tune the global model individually for each client [30].

### High Cost of Annotations

Supervised machine learning requires the annotation of radiology images before they can be used to train the model. Image-level annotations classify each image into one or more classes, whereas region-level annotations highlight regions within an image and classify each region into one or more classes. As the predictive performance of the model is directly influenced by the quality of the annotations, it is imperative that the data are annotated by qualified radiologists or medical practitioners [31]. This makes the process of annotation exorbitantly expensive in many cases.

Several efforts have been made to use natural language processing (NLP) techniques to automatically annotate images by extracting labels from radiology text reports [32-34]. Semisupervised approaches can be used when a small amount of labeled data are available along with a larger amount of unlabeled data [35,36]. As manual annotations are expensive, AI-based automated image annotation techniques can be considered [37].

### Ambiguous Ground Truth

As hospital data sets usually contain images accompanied by their text reports, many projects are kickstarted by using NLP techniques to automatically annotate the images using the reports. Radiology reports, however, vary widely in their comprehensiveness, style, language, and format [38]. Even if state-of-the-art NLP manages to accurately extract all the findings from the text report, the report itself may not mention all the findings. Olatunji et al [39] showed that there is a large discrepancy between what radiologists see in an image and what they mention in the report; reporting radiologists usually document only those findings that are relevant to the immediate clinical context and are likely to miss reporting nonactionable or borderline findings.

Radiology images suffer from significant interreader variability, where 2 or more experts may disagree on the findings from a scan [7,8,40-43]. Sakurada et al [44], for instance, report low interreader κ values ranging from 0.24 to 0.63 for assessment of different pathologies from chest radiographs. In practice, annotation workflows generally engage a single reader to assign ground truth labels to images. An improvement over this involves engaging multiple independent readers and considering their majority vote as the ground truth label. However, single reader or majority vote approaches may miss labeling challenging but critical findings.

This risk can be mitigated by using multiphase reviews [45] or expert adjudication [46] to create high-quality labels. Majkowska et al [46] showed that adjudication improved the consensus among radiologists to 96.8% compared with 41.8% after the first independent readings when assessing chest radiographs. Raykar et al [47] proposed a probabilistic approach to determine the *hidden* ground truth from labels assigned by multiple radiologists and demonstrated that this method is superior to majority voting. In some clinical settings, radiology imaging is used for initial screening before conducting subsequent confirmatory tests. For example, chest x-ray scans may be used as a first-line test before subsequently conducting a computed tomography scan, laboratory tests, or biopsy. Data from these subsequent tests, if available, should be used to validate and correct the labels assigned to the images from the screening test. In situations where human-labeled ground truth is noisy or ambiguous, developing a process to reduce variability and improve label quality may yield better models than attempts to improve model performance on the original labels by other means.

### Imbalance in Class Representation

Class imbalance occurs when all label classes are not equally represented in the training data set [48]. This is a common situation when building binary classifiers for medical data sets where the number of *normal* examples in which the target abnormality is absent is many times larger than the number of *abnormal* examples in which it is present. As machine learning models are usually trained by optimizing a loss function across all training examples, the trained models tend to favor the majority class over the minority class. Researchers have empirically evaluated the adverse effect of class imbalance on classification performance in several studies [9,49-53].

Class imbalance can be handled at the data level or the algorithmic level. Resampling strategies can be used to address imbalances in the training data by either undersampling the majority classes or oversampling the minority classes. Many comparative evaluations of these approaches exist, sometimes with contradictory conclusions. Drummond et al [54], for instance, argued that undersampling works better than oversampling, whereas Batista et al [55] reported superior performance using oversampling. However, we caution the reader against hasty generalizations and note that these comparisons are highly dependent on the data set, the machine learning algorithm, the sampling technique used, and the parameters of the experiments. Chawla et al [56] proposed the synthetic minority oversampling technique, a technique to generate synthetic examples to balance the data set, and showed that the combination of synthetic minority oversampling technique and undersampling performs better than plain undersampling or oversampling. Similarly, oversampling can also be performed using geometric augmentations, color-space augmentations, or generative models to produce synthetic images. An imbalance in the number of examples can also be addressed at the algorithmic level using methods such as one-class classification, outlier or anomaly detection, regularized ensembles, and custom loss functions [9,57-60].

### Asymmetric Misclassification Costs

Standard machine learning settings assume that all misclassifications between classes are equal and incur the same penalty. This assumption is not true for many medical imaging problems. For example, the cost of classifying a *normal* scan as *abnormal* may be very different from the cost of classifying an *abnormal* scan as *normal*.

This asymmetrical nature of the classification problem can be handled either at the time of deployment or during development. The trained model can be tuned to achieve higher sensitivity or specificity according to the requirements at deployment time. Alternatively, the variation in misclassification penalties can be represented as a cost matrix, where each element C(i,j) represents the penalty of misclassifying an example of class i as class j. The model can then be trained by minimizing the overall cost as defined by the asymmetrical loss function. For more details, we refer the reader to the literature on cost-sensitive learning [9,61,62].

### Relevant Performance Measures

Machine learning researchers and practitioners tend to ignore the question of how model performance should be evaluated in cases of imbalanced data sets and asymmetric misclassification costs. Most binary classification models produce a continuous-valued output score. This score is converted into discrete binary labels using a cutoff threshold. Owing to its simplicity, it is tempting to use accuracy, defined as the percentage of predictions that are correct, as a measure of performance. However, in the case of imbalanced data sets, accuracy is ineffective and provides an incomplete and often misleading picture of the ability of the classifier to discriminate between the two classes [63,64].

Using two or more measures such as sensitivity, specificity, and precision, provides a better picture of the discriminative performance of a classifier [65]. However, these measures depend on the cutoff threshold mentioned earlier. Furthermore, the decision to set the threshold is often guided not by technology but by business or domain concerns. Comparing the two models by considering multiple performance measures across different operating thresholds is challenging. The receiver operating characteristic curve, on the other hand, captures the model performance at all threshold operating points. The area under the receiver operating characteristic curve (AUROC) thus serves as a single numerical score that represents the performance of the model across all operating threshold points. This has made AUROC a metric of choice for reporting the classification performance of machine learning models. Unfortunately, AUROC too can be deceptive when dealing with imbalanced data sets and may provide an overly optimistic view of performance [9]. The precision-recall curve and the area under it are more suitable for describing classification performance when data sets are imbalanced [66,67]. Drummond and Holte proposed cost curves that describe the classification performance over asymmetric misclassification costs and class distributions [68,69]. Table 1 shows how accuracy can be misleading because of imbalanced data sets.

**Table 1:** Example illustrating how accuracy can be misleading in case of imbalanced data sets.

	Predicted as negative	Predicted as positive	Total
Actual negative	80	10	90
Actual positive	5	5	10
Total	85	15	100

In the confusion matrix mentioned earlier, of 100 test examples, 90 are negative and 10 are positive. The classifier predicts 85 of them as negative and 15 as positive. This gives a high accuracy of 0.85 and a high specificity of 0.89. However, the complete picture is seen when we consider the low sensitivity of 0.50 and precision of 0.33.

### Generalization of Models to Unseen Data Sets

Machine learning models are routinely evaluated on a hold-out set taken from the same source as the training set [70]. The available data are divided into two parts. One part is used to train and validate the models. The second part, called the test set or hold-out set, is used to estimate the final performance of the trained model when deployed. The underlying premise is that the data used to train the model are representative of the data that the model will encounter during clinical use. This assumption is often violated in practice, and this makes the performance on the hold-out set an unreliable indicator of future performance in clinical deployment.

Poor generalization of models to diverse patient groups is one of the biggest hurdles for the adoption of AI and machine learning in health care. One reason for the poor generalization is the difference in the image characteristics between images from the training sites and those from the deployment site. This variation, also known as data set shift, can occur because of differences in hospital procedures, equipment manufacturers, image acquisition parameters, disease manifestations, patient populations, among others. Owing to the data set shift, models trained using data from one hospital may perform poorly on data from another hospital [71]. We note here that this inability to generalize to data sets from an unseen origin is different from the problem of overfitting, where the model shows poor performance even on test sets from the same origin. Learning irrelevant confounders instead of relevant features is another reason why models fail to generalize to data from unseen origins. Machine learning models are notorious for exploiting confounders in the training data. For example, Zech et al [72] showed that a pneumonia classification model trained on data from 2 hospitals learned to leverage the difference between prevalence rates at the 2 hospitals instead of the relevant visual features.

Data augmentation can improve model generalization by increasing the variations in the training set [73]. Image processing techniques, including standardization, normalization, reorientation, registration, and histogram matching, can be used to harmonize images sourced from different origins and remove domain bias. However, Glocker et al [74] showed that even with a state-of-the-art image preprocessing pipeline, these techniques for harmonization were unable to remove scanner-specific bias, and machine learning models were easily able to discriminate between the different origins of the data.

Domain adaptation techniques can be used to fine-tune models to a new target domain by narrowing the gap between the source and target domains in a domain-invariant feature space [75-79]. On the other hand, domain generalization techniques attempt to train models that are sensitive only to features relevant to the classification task but insensitive to confounding features that differentiate between the domains [80-85].

### Model Decay

Model decay refers to the phenomenon in which the performance of a deployed machine learning model deteriorates over time [11]. Supervised machine learning algorithms extract patterns from the training data to learn a mapping between independent input variables and a dependent target variable. This process involves making an implicit assumption that the data encountered in deployment will be stationary and will not change over time; this assumption is often violated in practice because of the changes in hospital workflows, imaging equipment, patient groups, evolving adoption of AI solutions, among others.

Model decay occurs owing to changes in the underlying data. These changes can be broadly classified into three types: (1) *covariate shift* occurs when there are changes in the distribution of the independent input variables (eg, the average age of the population increases over time); (2) *prior probability shift* occurs when there are changes in the distribution of the dependent target variables (eg, the prevalence of a particular disease in the target population may change because of seasonality or an epidemic); and (3) *concept drift* occurs when there are changes in the relationship between the independent and dependent variables (eg, changes in a hospital’s diagnostic protocols or a radiologist’s interpretation regarding which visual manifestations should or should not be considered indicative of a pathology). These changes can be sudden, gradual, or cyclic.

Detecting model decay requires continuous monitoring of the deployment time performance against a human-labeled subsample of the data. If the performance drops below a predetermined threshold, an alarm is triggered, and the model is retrained or fine-tuned using the most recent data. This retraining can also be conducted periodically as a routine maintenance activity. For more details, including theoretical frameworks for understanding model decay or practical solutions, readers can refer to additional reviews [11,86-89].

### Adversarial Attacks

An adversarial example is constructed by deliberately injecting perturbations in the original image to trick the model into misclassifying the label for that image [12]. Machine learning models are susceptible to manipulation using such adversarial examples [90,91]. Data poisoning attacks [13] introduced adversarial examples in the training data to manipulate the diagnosis of the model being developed. On the other hand, evasion attacks [14] use adversarial examples to influence predictions during deployment. Health care is a huge economy, and many decisions regarding diagnosis, reimbursements, and insurance may be governed or assisted by algorithms in the near future. Hence, the discovery of these vulnerabilities has raised pressing concerns regarding the safety and usability of machine learning models in clinical practice.

Qayyum et al [92] provided a detailed taxonomy of defensive techniques against adversarial attacks by grouping them into three broad categories: (1) reconstructing the training or testing data to make it more difficult to manipulate [90,93-96], (2) modifying the model to make it more resilient to adversarial examples [97-101], and (3) using auxiliary models or ensembles to detect and neutralize adversarial examples [102-106]. Adversarial attacks and their countermeasures are an evolving research area, and there are excellent reviews for the same [107-109].

### Explainability

The power of neural networks to uncover hidden relationships between variables and use them to make predictions is tempered by one disadvantage: the exact process the neural network uses to arrive at a decision is unclear to humans. This is why neural networks are sometimes called black boxes whose inner workings cannot be observed. To what extent can we delegate decision-making to machines while we remain unaware of how the machine arrives at a decision is a key question that stands in the way of adopting algorithms in many industries, including autonomous vehicles, law, finance, among others.

Algorithmic explainability is especially important in medicine, where stakes are high, and the field is prone to litigation. In the context of radiology, explainability can be improved by using localization models that highlight the region of interest within the scan that is suspected to contain the abnormality instead of classification models that only indicate the presence or absence of an abnormality. However, the development of localization models also requires training data to have region-based annotations in the form of bounding boxes or free-form masks. Where region-based annotations are not available, saliency maps [110] and explainability frameworks [111] can be used to identify a region within the image that most contributes to a particular decision. Another way to improve the user’s trust in the models is to predict a confidence score in addition to the prediction. For example, instead of merely stating the prediction “Probability of Tuberculosis: 75%,” the system should also state the model’s confidence “Probability of Tuberculosis: 75%, Confidence in this prediction: Low.” Deployment settings where predictive models are used to autonomously make decisions demand more stringent conditions of explainability than settings where the models are used to guide humans who make the final decisions. A comprehensive analysis of explainers in the domain of computer vision was performed by Buhrmester et al [112].

There have been calls to limit the use of AI and machine learning only to rule-based systems in fields where algorithmic decisions affect human lives [113]. These systems are transparent and can trace the relationship between the input and the output as a sequence of rules that humans can understand. We find two problems with this approach. First, one of the chief advantages of using neural networks is that they can model complex relationships that *humans cannot understand*, and this is precisely what makes them so effective. Second, making decision systems transparent and explainable also makes them vulnerable to malicious attacks. A transparent rule-based method to make decisions can be *hacked*, *gamed*, or exploited more easily than a black box system [114,115].

### Fairness and Bias

Algorithmic systems play a key role in guiding decisions that impact the delivery of health care to patients. Therefore, it is desirable that these systems are free of societal biases and their decisions are fair and equitable. Unfortunately, many existing data sets [18,43] reflect the biases of the societies that they represent [116], and it is difficult to detect and remove bias inherent in the training data. Obermeyer et al [16] showed, for example, that a widely used algorithmic system exhibited racial bias against Black patients, which reduced the number of Black patients eligible for extra care by more than half.

In principle, a predictive model is considered fair if it does not discriminate patients on the basis of sensitive variables such as gender, ethnicity, disability, and income. However, translating this seemingly simple principle into practice is a challenging issue. Researchers have developed numerous mathematical definitions of fairness and techniques to implement them [117]. One technique, for example, excludes sensitive variables from the input when training the model. Another technique is to tune the model so that it demonstrates the same level of performance as measured by sensitivity, specificity, among others, across all groups defined by the sensitive variables. Corbett-Devies and Goel [118] show that although appealing, these techniques suffer from significant statistical limitations and may adversely affect the same groups they were designed to protect. Pleiss et al [119] show how different definitions of fairness can be mutually incompatible, and a model designed to comply with one definition may violate another equally valid definition.

Algorithmic bias and fairness are evolving fields of research that lie at the intersection of machine learning, public policy, law, and ethics. We believe that fairness is not inherently a technological problem but a societal one. Coercing technology to solve it can lead to automated systems that tick the right boxes for some arbitrary definition of fairness but eventually end up worsening social inequality and discrimination behind a veneer of technical neutrality [120].

### Clinical Validation

A comprehensive evaluation to assess the predictive performance and clinical utility of a model must be conducted before it can be deployed in clinical practice. When a model is evaluated on a hold-out set collected from the same sources from which the training data are collected, the evaluation is called an *internal* validation. When a data set from an *unseen* source is used to evaluate the model, the evaluation is called *external* validation. As described earlier in the section *Generalization of Models to Unseen Data Sets*, the lack of generalization to unseen data sources is one of the biggest challenges in the adoption of machine learning in practice. Despite this, only a fraction of the published studies report the results of an external validation [121]. Mahajan et al [122] presented examples to advocate the case for independent external validation of models before deployment and described a framework for the same. Park et al [31] proposed a methodology with a checklist for evaluating the clinical performance of the models. The TRIPOD statement [123] provides guidelines for transparent reporting of the development and validation of prediction models for prognosis and diagnosis models. Although retrospective evaluations allow machine learning developers to test their models on large and diverse data sets, prospective evaluations allow testing in real-world environments; both types of evaluations are equally important and should be meticulously carried out before full-scale adoption.

## Conclusions

We identify the key challenges that researchers face in developing accurate, robust, and usable machine learning models that can create value in clinical radiology practice. These challenges and the techniques to overcome them have been discussed previously in a piecemeal manner in prior research literature. In this study, we re-examined them in the context of medical imaging. By compiling them in the form of a laundry list, we hope to make this research more readily accessible.

Hospital workflows and practices vary widely from one hospital to another, even within the same geography. This increases the difficulty of seamlessly integrating predictive models into hospital workflows. The nonuniformity in workflows also raises the question of whether the reported performance of a model is reproducible in a different clinical context. This is an ongoing research, and satisfactory solutions are yet to be found.

The ultimate objective of diagnostic machine learning models is to improve patient outcomes. However, improvement in diagnostic performance does not, by itself, cause an improvement in patient outcomes [31]. Radiological diagnosis is only one of the many steps that eventually leads to treatment. Therefore, a computerized diagnostic system must be placed appropriately in the workflow. How the system presents the results to the reporting radiologist and what action the radiologist takes on receiving them are important factors that influence the usefulness of the system in practice.

On the one hand, medical imaging is a broad and complex field that encompasses numerous imaging modalities, pathological conditions, and diagnostic protocols. On the other hand, machine learning is an active area of research with thousands of new techniques published every year. The combined diversity of both fields along with nonuniform hospital practices, regulatory restrictions on data sharing, and lack of standardized reporting of results make it difficult to clearly assess the role and potential of machine learning applications in medical imaging. We believe that machine learning has great potential in improving diagnostic accuracy, lowering reporting times, reducing radiologist workloads, and ultimately improving the delivery of health care. To realize this potential, however, a concerted across-the-board effort will be required from physicians, radiologists, patients, hospital administrators, data scientists, software developers, and other stakeholders.

## Abbreviations

AI: artificial intelligence
AUROC: area under the receiver operating characteristic curve
NLP: natural language processing
